# Correlates of burnout among Chinese ICU nurses: a meta-analysis

**DOI:** 10.3389/fpubh.2026.1763539

**Published:** 2026-07-13

**Authors:** Qiujie Wang, Qiong Chai, Zhanxin Fan

**Affiliations:** College of Nursing and Health, Henan University, Kaifeng, China

**Keywords:** burnout, China, intensive care unit, meta-analysis, nurse

## Abstract

**Background:**

Burnout is prevalent among nurses, particularly in Intensive Care Units (ICUs), where work is characterized by high intensity, high risk, and heavy workloads. This condition not only directly impairs nurses’ physical and mental health and reduces their occupational retention rate but may also threaten the safety of ICU patients due to potential declines in the quality of nursing care. Given these implications, effective alleviation of burnout among ICU nurses is essential for promoting their well-being, stabilizing the nursing workforce, and improving patient satisfaction. Previous studies have explored various variables related to burnout among ICU nurses, such as psychological capital, social support, work–family conflict, and moral distress. However, these findings remain inconsistent and fragmented across studies. To date, a comprehensive quantitative synthesis of the correlations between these variables and burnout among ICU nurses is lacking. Therefore, this study conducted a meta-analysis based on Pearson correlation coefficients to examine the correlates of burnout among ICU nurses, aiming to provide more reliable evidence for the development of targeted intervention strategies for this population.

**Methods:**

Databases including CNKI, Wanfang Database, VIP Database, China Biomedical Literature Database (CBM), PubMed, Web of Science, Embase, CINAHL, and Cochrane Library were systematically searched. The search used terms for intensive care units, nurses, burnout, and China in both Chinese and English. The search was conducted from the inception of the database to September 2025. Two researchers with expertise in evidence-based practice performed the literature search, independently screened studies based on the inclusion and exclusion criteria, assessed the quality of the included studies using the Agency for Healthcare Research and Quality (AHRQ)’s quality assessment criteria for observational studies, and extracted relevant data. A meta-analysis was conducted using RevMan 5.4 software, with Pearson correlation coefficients (r values) as the outcome indicator.

**Results:**

A total of 31 studies were included, with a combined sample size of 8,219 participants, and the overall methodological quality of the included studies was moderate. The pooled analysis indicated that burnout was significantly negatively correlated with psychological capital (*r* = −0.43, 95% CI: −0.53 to −0.33, *I*^2^ = 81%), research competence (*r* = −0.21, 95% CI: −0.30 to −0.11, *I*^2^ = 0%), and social support (*r* = −0.40, 95% CI: −0.49 to −0.28, *I*^2^ = 82%) (*p* < 0.01). Burnout was positively correlated with work–family conflict (*r* = 0.49, 95% CI: 0.41–0.57, *I*^2^ = 69%), presenteeism (*r* = 0.33, 95% CI: 0.24–0.41, *I*^2^ = 0%), moral distress (*r* = 0.32, 95% CI: 0.16–0.46, *I*^2^ = 53%), turnover intention (*r* = 0.48, 95% CI: 0.24–0.66, *I*^2^ = 96%), and alarm fatigue (*r* = 0.55, 95% CI: 0.26–0.75, *I*^2^ = 95%) (*p* < 0.01). Substantial heterogeneity was observed in several pooled estimates, particularly for turnover intention and alarm fatigue.

**Conclusion and recommendations:**

This meta-analysis suggests that burnout among ICU nurses is associated with multiple occupational and psychosocial factors, including work–family conflict, turnover intention, presenteeism, social support, and psychological capital. These findings highlight the importance of work-related stressors and psychological resources in relation to burnout among nurses. However, given the heterogeneity across studies and the cross-sectional nature of the included evidence, these associations should be interpreted with caution. The results provide a synthesized overview of the factors related to burnout among ICU nurses and may help inform future research and the development of strategies aimed at supporting nurses’ occupational well-being.

**Systematic review registration:**

This study was registered in the PROSPERO database (registration number: CRD420251146719). The protocol predefined the research objectives, inclusion and exclusion criteria, and main analytical methods. This study was conducted in accordance with the registered protocol. If minor adjustments were made during the study due to data availability, they were clearly described and justified in the Methods section to improve transparency and reproducibility.

## 1 Introduction

Job burnout is a state of physical and psychological exhaustion resulting from prolonged exposure to work-related stress and the depletion of personal and organizational resources. It is typically characterized by three core dimensions: emotional exhaustion, depersonalization, and reduced personal accomplishment (1). In recent years, nurse burnout has been increasingly recognized as a major challenge for healthcare systems and nursing management worldwide. Previous systematic reviews and meta-analyses have reported substantial levels of burnout among nurses across different countries and healthcare settings (2, 3). However, estimates of burnout prevalence vary considerably depending on the measurement instruments used, cutoff criteria applied, and specific burnout dimensions assessed. For example, a meta-analysis covering 49 countries reported an overall prevalence of approximately 11.23% among nurses (3), although such estimates should be interpreted cautiously given the methodological heterogeneity across studies.

Nurses working in intensive care units (ICUs) are particularly vulnerable to burnout due to their high workload, high-risk clinical tasks, and high-intensity work environments (4). ICU nurses are required to continuously monitor critically ill patients, respond rapidly to complex emergencies, and cope with multiple stressors such as patient deterioration, death, and the expectations of patients’ families. These demanding job characteristics make ICU nurses a population at a high risk of burnout. A previous study (5) suggested that ICU nurses may experience higher levels of burnout than those working in non-ICU settings. Burnout not only affects nurses’ physical and mental health and increases turnover intention but may also be associated with declines in care quality and a higher risk of medical errors, thereby potentially threatening patient safety and healthcare quality (6, 7).

Identifying the factors associated with ICU nurses’ burnout is therefore important for understanding the mechanisms underlying burnout, informing future research, workforce management, and targeted intervention strategies. Previous studies (8–10) have examined the roles of psychological capital, social support, organizational commitment, emotional labor, and other related factors in ICU nurses’ burnout. However, the existing evidence remains inconsistent and fragmented. This fragmentation may be partly explained by differences in ICU settings, nurse populations, burnout measurement instruments, burnout dimensions, and statistical methods used across studies. For example, previous studies have been conducted in different types of ICUs, including general, emergency, and specialized ICUs (11, 12), where workload intensity, patient acuity, staffing patterns, and organizational support may vary substantially. Additionally, the characteristics of nurse populations differ across studies in terms of age, years of ICU experience, professional title, shift work, and employment status. These differences may contribute to inconsistent findings regarding the factors associated with burnout.

Moreover, methodological differences across studies limit the comparability of existing findings. Some studies reported total burnout scores, whereas others separately reported specific dimensions such as emotional exhaustion, depersonalization, or reduced personal accomplishment (8, 9). Differences in measurement instruments and scoring methods may also influence the estimated strength and direction of the associations between related factors and burnout. Furthermore, statistical reporting varied across studies; for example, some studies used different types of correlation coefficients or reported only dimension-specific burnout scores (8, 13), which increases the difficulty of quantitative synthesis. Most previous studies were single-center cross-sectional investigations (9, 10) with relatively limited sample sources and study contexts. Such designs may not fully reflect the overall characteristics of burnout-related factors among Chinese ICU nurses and may further limit the generalizability and comparability of the existing findings. Therefore, it remains unclear which factors show relatively stable associations with burnout among ICU nurses and which findings may be influenced by the study context or sample characteristics.

Focusing specifically on Chinese ICU nurses is necessary because burnout and its related factors may be shaped by specific healthcare and sociocultural contexts. In China, ICU nurses may experience context-specific occupational pressures related to high workload intensity, rotating shifts, staffing constraints, hierarchical clinical environments, and cultural expectations regarding work and family responsibilities. These contextual factors may influence both the level of burnout and its associations with psychological, organizational, and social factors. Therefore, synthesizing evidence from Chinese ICU nurses may provide more context-specific implications for burnout prevention, nursing workforce management, and quality improvement in critical care settings.

Accordingly, this study conducted a meta-analysis based on the Pearson correlation coefficients reported in the existing literature to systematically synthesize and quantify the associations between job burnout and related factors among Chinese ICU nurses. By focusing on Pearson correlation coefficients and total burnout scores, this study aimed to standardize the effect size estimation and reduce methodological heterogeneity arising from differences in statistical reporting and burnout outcome definitions across studies. The methodological contribution of this study lies in integrating scattered correlational findings, improving the comparability and interpretability of pooled estimates, and identifying relatively stable burnout-related factors in this specific population. These findings may provide evidence for developing targeted intervention strategies and guiding future research on burnout prevention among Chinese ICU nurses.

## 2 Methods

### 2.1 Eligibility criteria

Inclusion criteria: (1) The study population consisted of ICU nurses in China. (2) The study design was cross-sectional; therefore, the findings were interpreted as correlational associations only, without implications for causal relationships or temporal order. (3) Burnout was measured using the Maslach Burnout Inventory (MBI) or its derivative scales, such as the MBI-HSS or MBI-GS, with acceptable reliability reported in the original study. (4) Eligible studies reported Pearson correlation coefficients between total burnout scores and related factors or provided sufficient data to extract effect sizes suitable for quantitative synthesis.

Exclusion criteria: (1) Studies published in languages other than Chinese or English. (2) Duplicate publications. (3) Studies for which the full text was unavailable or the data were incomplete. (4) Conference papers, abstracts, reviews, editorials, or commentaries. (5) Studies that did not report correlation coefficients suitable for meta-analysis (e.g., descriptive studies, normative comparisons, or studies reporting only group differences without correlation coefficients). (6) Studies reporting only dimension-level burnout correlations, such as emotional exhaustion, depersonalization, or reduced personal accomplishment, without providing total burnout scores. (7) Studies reporting only Spearman correlation coefficients or other statistical measures that could not be directly synthesized or converted into Pearson correlation coefficients. (8) Studies with low methodological quality.

To ensure statistical and conceptual consistency, this meta-analysis included studies reporting Pearson correlation coefficients between total burnout scores and related factors. Studies reporting only Spearman correlation coefficients were excluded because the Spearman coefficient is a rank-based measure and is not statistically equivalent to Pearson’s r. Studies reporting only dimension-level burnout outcomes were also excluded because burnout dimensions represent distinct aspects of the construct, and combining them with total burnout scores may introduce conceptual inconsistency and potential double counting.

### 2.2 Design and search strategy

A systematic search was conducted in PubMed, Web of Science, Embase, CINAHL, Cochrane Library, CNKI, Wanfang Database, VIP Database, and Chinese Biomedical Literature Database (CBM). The search was conducted from database inception to September 2025. Both subject headings and free-text terms were used in combination, and the reference lists of all included studies were manually screened to identify additional relevant articles. The main search terms included ‘Intensive Care Unit/Unit, Intensive Care/ICU/Intensive Care Units’; ‘Burnout, Professional/burnout/job burnout/career burnout/occupational burnout’; ‘nurse/nursing/nursing staff’; and ‘China/Taiwan/Hong Kong/Macau.’ The PubMed search strategy was as follows:

#1: “Intensive Care Units”[Mesh] OR “Intensive Care Unit”[Title/Abstract] OR “Unit, Intensive Care”[Title/Abstract] OR ICU[Title/Abstract].

#2: “Burnout, Professional”[Mesh] OR Burnout[Title/Abstract] OR “Job Burnout”[Title/Abstract] OR “Career Burnout”[Title/Abstract] OR “Occupational Burnout”[Title/Abstract].

#3: “Nurses”[Mesh] OR nurse[Title/Abstract] OR nursing[Title/Abstract] OR “nursing staff”[Title/Abstract].

#4: China[Title/Abstract] OR Taiwan[Title/Abstract] OR “Hong Kong”[Title/Abstract] OR Macau[Title/Abstract].

#5: #1 and #2 and #3 and #4.

### 2.3 Literature screening and data extraction

Duplicate records were removed using EndNote 21, followed by a manual check to eliminate any remaining duplicates. Screening was conducted in three stages. First, two reviewers independently screened the titles and abstracts to exclude irrelevant records. Second, the full texts of potentially relevant studies were retrieved and assessed according to the eligibility criteria. Finally, studies that met the inclusion criteria were included for data extraction and further analysis. During the selection process, any disagreements between the two reviewers were resolved through discussion. If consensus could not be reached, a third reviewer was consulted, and a final decision was made. Data extracted from each included study comprised: (1) basic study information—title, first author, year of publication, burnout assessment instrument, and related factors; (2) characteristics of the study population—geographic region, sample size, and sampling method; and (3) Pearson correlation coefficients between total burnout scores and each related factor, sample size, and other data required for effect size calculation.

### 2.4 Literature quality assessment

The quality of the studies was independently assessed by two researchers with systematic training in evidence-based nursing. In case of disagreement, the two reviewers reached a consensus, or a third reviewer was consulted to make a final decision. Study quality was evaluated using the Agency for Healthcare Research and Quality (AHRQ) checklist for observational studies (14). This tool includes 11 items: source of data, inclusion and exclusion criteria, time period of the study population, continuity of study subjects, characteristics of study subjects, reassessment, reasons for excluding subjects from analysis, methods for controlling confounding factors, handling of missing data, response rate and completeness of data collection, and follow-up. Each item is scored as ‘Yes’ (1 point), ‘No’ (0 points), or ‘Unclear’ (0 points), with a total score ranging from 0 to 11. Studies scoring 0–3 were considered low quality, 4–7 moderate quality, and 8–11 high quality. Low-quality studies were excluded from this systematic review and meta-analysis.

### 2.5 Data analysis methods

The meta-analysis was performed using RevMan 5.4 software. Pearson correlation coefficients were used as the effect size for the associations between job burnout and related factors. Before pooling, the original correlation coefficients (r values) were transformed into Fisher’s *Z* values because the raw correlation coefficients were not normally distributed, particularly when they approached −1 or 1. Fisher’s Z transformation stabilizes the variance and makes the effect sizes more suitable for quantitative synthesis. The corresponding standard errors (SEs) were calculated according to the established formula (15). After pooling, the combined Fisher’s *Z* values and 95% confidence intervals (CIs) were transformed back into correlation coefficients (r values) for interpretation.

The pooled Fisher’s *Z* values were estimated using the inverse variance method. This approach assigned greater weight to studies with smaller standard errors and more precise estimates, thereby improving the statistical efficiency of the pooled results. Considering the potential methodological and clinical heterogeneity across studies, including differences in ICU settings, nurse characteristics, measurement instruments, and burnout-related factors, a random-effects model using the DerSimonian–Laird method was primarily applied. This model accounts for both within-study sampling errors and between-study variability. Fixed-effect models were used for analyses with negligible heterogeneity. The *I*^2^ statistic was used to assess heterogeneity; however, it was not the sole criterion for model selection.

To ensure consistency in the interpretation of effect directions, the scoring direction of all included scales was carefully examined and, where necessary, adjusted to maintain a consistent direction of effects across studies. A descriptive analysis was conducted for factors with a small number of studies or insufficient data for pooling. Additionally, the results were interpreted with a focus on the magnitude and precision of effect sizes, namely the pooled r values and 95% CIs, rather than relying solely on statistical significance.

As this meta-analysis synthesized the correlation coefficients derived from cross-sectional studies, the pooled results should be interpreted as the direction and strength of the associations between job burnout and related factors. They do not indicate causal relationships or temporal order. Therefore, these findings should be interpreted as correlational evidence that may inform future longitudinal studies and intervention research.

## 3 Results

### 3.1 Literature search results

**Figure 1:**
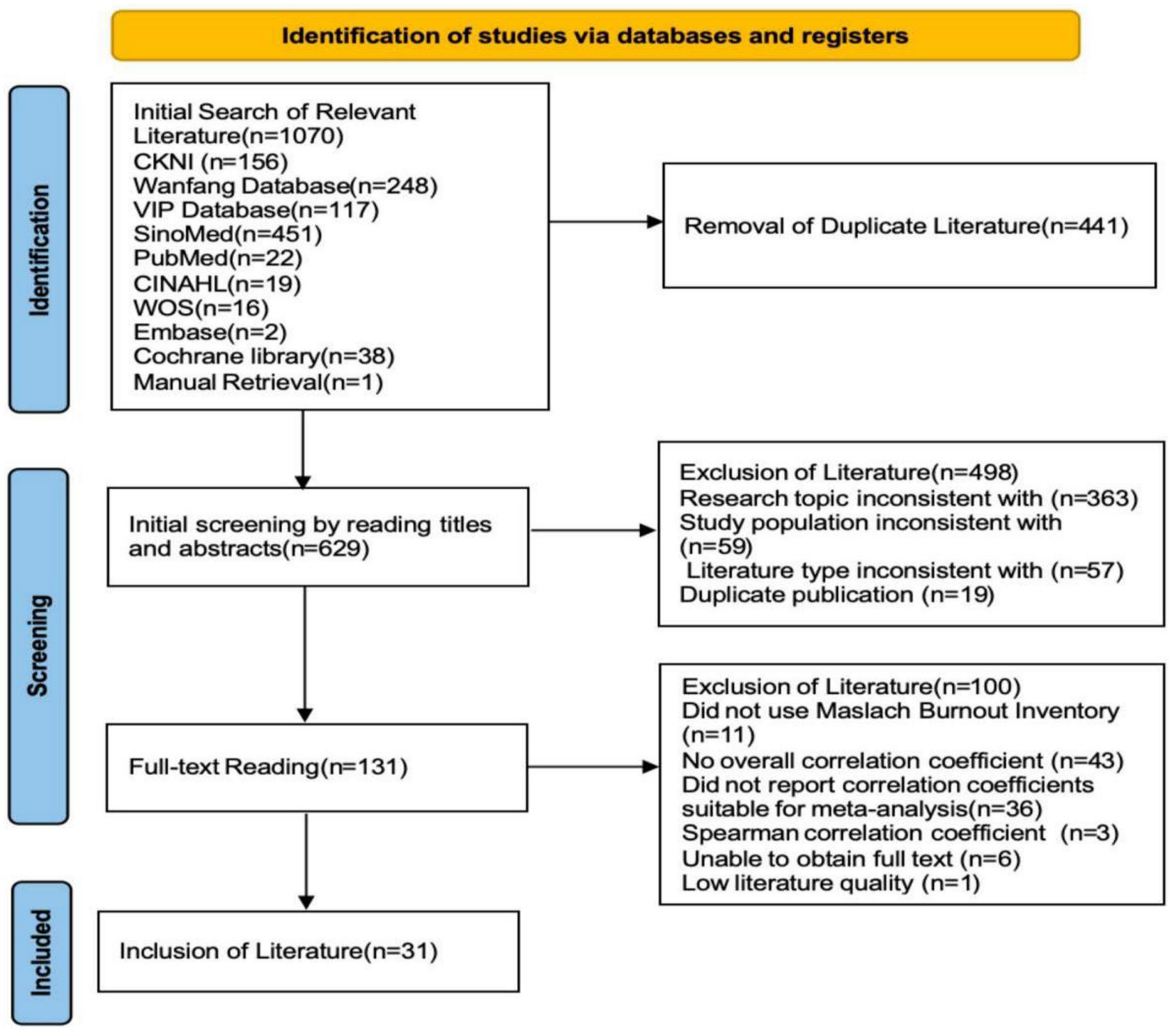
PRISMA flow diagram of the selection process of studies.

A systematic search of CNKI, Wanfang Database, VIP Database, Chinese Biomedical Literature Database (CBM), PubMed, Web of Science, Embase, CINAHL, and the Cochrane Library yielded 1,070 records. After removing 441 duplicates, 498 records were excluded based on title and abstract screening. Following a full-text review and quality assessment, 100 articles were excluded. Ultimately, 31 studies (9, 10, 16–44) were included in the meta-analysis (Figure 1).

### 3.2 Characteristics of included studies and results of study quality assessment

A total of 31 studies were included, with a combined sample size of 8,219. All studies used the Maslach Burnout Inventory (MBI) or its derivative scales with acceptable reliability. The quality assessment scores of the included studies ranged from 4 to 8, suggesting that all studies were of moderate or higher quality. The basic characteristics of the included studies are shown in Table 1, and the quality assessment results are presented in Table 2.

**Table 1:** Basic characteristics of the included literature.

First author/Year	Sample size	Sampling methods	Region	Evaluation tools	Relevant factors
Yao 2022 (9)	82	Convenient sampling	Henan	MBI-GS	A, B
Du 2022 (10)	1,114	Convenient sampling	Jiangxi	MBI	C
Jiang 2020 (16)	273	Convenient sampling	Jiangsu	MBI-GS	A
Zheng 2021 (17)	198	Convenient sampling	Henan	MBI-GS	A
Lin 2017 (18)	203	Convenient sampling	Fujian	MBI	C
Wu 2023 (19)	474	Convenient sampling	Guangdong	MBI	D, E
Ma 2022 (20)	183	Convenient sampling	Shandong	MBI	D
Xue 2022 (21)	203	Random sampling	Hangzhou	MBI-GS	C, D
Ma 2016 (22)	182	Convenient sampling	Hubei	MBI	A
Xia 2019 (23)	300	Convenient sampling	Guangxi	MBI	A, F
Zhang 2024 (24)	386	Convenient sampling	Jiangxi	MBI	A, C, G
Tang 2020 (25)	60	Convenient sampling	Guangdong	MBI	A
Liu 2019 (26)	200	Convenient sampling	Beijing	MBI	H
Liang 2017 (27)	210	Convenient sampling	Qingdao	MBI	H
Ding 2025 (28)	255	Convenient sampling	Jiangsu	MBI	I, J, K
Liu 2024 (29)	332	Convenient sampling	Liaoning	MBI-GS	I, L
Zhang 2014 (30)	105	Convenient sampling	Nanchang	MBI	M
Yu 2022 (31)	222	Convenient sampling	Shandong	MBI	M
Lin 2024 (32)	358	Convenient sampling	Jiangsu	MBI	N, O
Ma 2023 (33)	683	Convenient sampling	National	MBI-GS	P, Q
Li 2023 (34)	412	Convenient sampling	Shandong	MBI	G, R
Li 2024 (35)	312	Cluster sampling	Hubei	MBI	G, S
Liu 2023 (36)	217	Convenient sampling	Sichuan	MBI	G
Fan 2014 (37)	181	Convenient sampling	Shandong	MBI	T
Ding 2015 (38)	56	Convenient sampling	Xinjiang	MBI	T
Gao 2015 (39)	181	Convenient sampling	Shandong	MBI	T
Liu 2012 (40)	169	Cluster sampling	Shandong	MBI	U
Zhu 2020 (41)	193	Convenient sampling	Jining	MBI	V
Lv 2020 (42)	50	Convenient sampling	Guangdong	MBI	W
Liu 2016 (43)	233	Cluster sampling	Shandong	MBI	X
Kadireyaguli 2025 (44)	192	Random sampling	Xinjiang	MBI	D

A: Psychological capital; B: Professional Benefit Perception; C: Social support; D: Work–family conflict; E: Job Engagement; F: Job Performance; G: Turnover Intention; H: Presenteeism; I: Alarm Fatigue; J: Workload; K: Missed Nursing Care; L: Job Satisfaction; M: Moral Distress; N: Perceived Ethical Climate; O: Workplace Spirituality; P: Emotional Intelligence; Q: Perceived Stress; R: Emotional Labor; S: Rights Protection; T: Research Competence; U: Work Stress; V: Work-Life Quality; W: Compassion Fatigue; X: Organizational Commitment.

**Table 2:** Results of literature quality evaluation.

First author/Year	Q1	Q2	Q3	Q4	Q5	Q6	Q7	Q8	Q9	Q10	Q11	Score	Literature level
Yao 2022 (9)	Yes	Yes	Yes	Yes	No	No	No	Yes	No	Yes	No	6	Medium
Du 2022 (10)	Yes	Yes	Yes	Yes	No	Yes	Yes	No	No	Yes	No	7	Medium
Jiang 2020 (16)	Yes	Yes	Yes	Yes	No	Yes	No	No	No	Yes	No	6	Medium
Zheng 2021 (17)	Yes	Yes	Yes	Yes	No	Yes	No	Yes	No	Yes	No	7	Medium
Lin 2017 (18)	Yes	Yes	Yes	Yes	No	No	Yes	Yes	No	Yes	No	7	Medium
Wu 2023 (19)	Yes	Yes	Yes	Yes	Unclear	No	Yes	No	No	Yes	No	6	Medium
Ma 2022 (20)	Yes	Yes	Yes	Yes	No	No	No	Yes	No	Yes	No	6	Medium
Xue 2022 (21)	Yes	Yes	Yes	Yes	No	Yes	No	No	No	Yes	No	6	Medium
Ma 2016 (22)	Yes	Yes	Yes	Yes	No	Yes	No	No	No	Yes	No	6	Medium
Xia 2019 (23)	Yes	Yes	Yes	Yes	No	Yes	Yes	No	No	Yes	No	7	Medium
Zhang 2024 (24)	Yes	Yes	Yes	Yes	No	Yes	Yes	No	No	Yes	No	7	Medium
Tang 2020 (25)	Yes	Yes	Yes	Yes	No	Yes	No	No	No	Yes	No	6	Medium
Liu 2019 (26)	Yes	Yes	Yes	Yes	No	Yes	No	Yes	No	Yes	No	7	Medium
Liang 2017 (27)	Yes	Yes	Yes	Yes	Unclear	No	Yes	Yes	No	Yes	No	7	Medium
Ding 2025 (28)	Yes	Yes	Yes	Yes	No	Yes	Yes	No	No	Yes	No	7	Medium
Liu 2024 (29)	Yes	Yes	Yes	Yes	No	Yes	No	No	No	Yes	No	6	Medium
Zhang 2014 (30)	Yes	Yes	Yes	Yes	No	No	No	No	No	Yes	No	5	Medium
Yu 2022 (31)	Yes	Yes	Yes	Yes	No	No	No	No	No	Yes	No	5	Medium
Lin 2024 (32)	Yes	Yes	Yes	Yes	No	Yes	No	Yes	No	Yes	No	7	Medium
Ma 2023 (33)	Yes	Yes	Yes	Yes	Unclear	Yes	Yes	No	No	Yes	No	7	Medium
Li 2023 (34)	Yes	Yes	Yes	Yes	No	No	No	No	No	Yes	No	5	Medium
Li 2024 (35)	Yes	Yes	Yes	Yes	Unclear	Yes	No	No	No	Yes	No	5	Medium
Liu 2023 (36)	Yes	Yes	Yes	Yes	No	Yes	Yes	Yes	No	Yes	No	8	High
Fan 2014 (37)	Yes	Yes	Yes	Yes	No	No	No	No	No	Yes	No	5	Medium
Ding 2015 (38)	Yes	Yes	Yes	Yes	No	No	No	No	No	Yes	No	5	Medium
Gao 2015 (39)	Yes	Yes	Yes	Yes	No	No	No	No	No	Yes	No	5	Medium
Liu 2012 (40)	Yes	No	Yes	Yes	No	No	No	No	No	Yes	No	4	Medium
Zhu 2020 (41)	Yes	Yes	Yes	Yes	No	No	Yes	No	No	Yes	No	6	Medium
Lv 2020 (42)	Yes	Yes	Yes	Yes	No	Yes	No	No	No	Yes	No	5	Medium
Liu 2016 (43)	Yes	Yes	Yes	Yes	No	Yes	No	No	No	Yes	No	6	Medium
Kadireyaguli 2025 (44)	Yes	Yes	Yes	Yes	No	Yes	No	No	No	Yes	No	6	Medium

Q1: Sources of data; Q2: Eligibility criteria; Q3: Time limit of the study population; Q4: Continuity of the study subjects; Q5: Other circumstances of the research subjects; Q6: Reassessment; Q7: Rationale for exclusion from analysis; Q8: Control measures for confounding factors; Q9: Handling of lost data; Q10: Response Rate and Completeness of Data Collection; Q11: Follow-up.

### 3.3 Results of the meta-analysis of burnout and associated factors

Pearson correlation coefficients (r values) related to the outcome variable were extracted from the 31 included studies, and the corresponding Fisher’s *Z* values and standard errors (SE) were calculated and entered into RevMan 5.4. A total of 24 associated factors were extracted. Meta-analyses were conducted for eight factors with data from two or more studies (Table 3), while the remaining 16 factors were assessed using a descriptive analysis (Table 4).

**Table 3:** Eight associated factors.

No.	Associated factors	Fisher’s *Z* and 95% CI	Summary *r* and 95% CI
1	Psychological capital	−0.46 (−0.59, −0.34)	−0.43 (−0.53, −0.33)
2	Social support	−0.42 (−0.54, −0.29)	−0.40 (−0.49, −0.28)
3	Work–family conflict	0.54 (0.43,0.65)	0.49 (0.41, 0.57)
4	Turnover intention	0.52 (0.24,0.80)	0.48 (0.24, 0.66)
5	Presenteeism	0.34 (0.24,0.44)	0.33 (0.24, 0.41)
6	Alarm fatigue	0.62 (0.27,0.97)	0.55 (0.26, 0.75)
7	Moral distress	0.33 (0.16,0.50)	0.32 (0.16, 0.46)
8	Research competence	−0.21 (−0.31, −0.11)	−0.21 (−0.30, −0.11)

**Table 4:** Descriptive summary of factors reported in fewer than two studies.

No.	First author	Year	Correlation coefficient (*r*)	*n* (studies)	Associated factors
1	Yao J	2022	−0.624	1	Professional Benefit Perception
2	Wu SH	2023	−0.400	1	Job Engagement
3	Xia HL	2019	−0.248	1	Job Performance
4	Ding R	2025	0.761	1	Missed Nursing Care
5	Ding R	2025	0.736	1	Workload
6	Liu FL	2024	−0.561	1	Job Satisfaction
7	Lin SQ	2024	−0.509	1	Perceived Ethical Climate
8	Lin SQ	2024	−0.551	1	Workplace Spirituality
9	Ma XC	2023	−0.465	1	Emotional Intelligence
10	Ma XC	2023	0.543	1	Perceived Stress
11	Li W	2023	0.519	1	Emotional Labor
12	Li L	2024	−0.200	1	Rights Protection
13	Liu Y	2012	0.470	1	Work Stress
14	Zhu LN	2020	−0.482	1	Work-Life Quality
15	Lv JY	2020	0.398	1	Compassion Fatigue
16	Liu Y	2016	−0.22	1	Organizational Commitment

#### 3.3.1 Correlation between burnout and psychological capital

**Figure 2:**
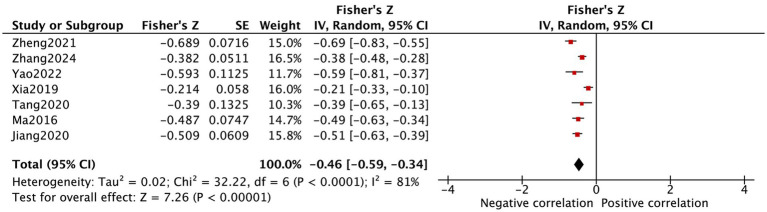
Fisher’s *Z* forest plot for burnout and psychological capital.

Figure 2 shows the pooled results for the association between burnout and psychological capital. The pooled Fisher’s *Z* value was −0.46 (95% CI: −0.59 to −0.34), corresponding to a correlation coefficient of *r* = −0.43 (95% CI: −0.53 to −0.33), indicating a moderate negative association between burnout and psychological capital.

Substantial heterogeneity was observed across the studies (*I*^2^ = 81%), suggesting considerable variability. Potential sources of heterogeneity will be further explored in subsequent subgroup analyses.

#### 3.3.2 Correlation between burnout and social support

**Figure 3:**
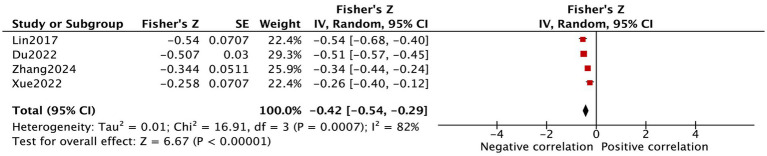
Fisher’s *Z* forest plot for burnout and social support.

Figure 3 shows the pooled results for the association between burnout and social support. The pooled Fisher’s *Z* value was −0.42 (95% CI: −0.54 to −0.29), corresponding to a correlation coefficient of *r* = −0.40 (95% CI: −0.49 to −0.28), indicating a moderate negative association between burnout and social support.

Substantial heterogeneity was observed across the studies (*I*^2^ = 82%), suggesting considerable variability. The potential sources of heterogeneity will be further explored in subsequent subgroup analyses.

#### 3.3.3 Correlation between burnout and work–family conflict

**Figure 4:**
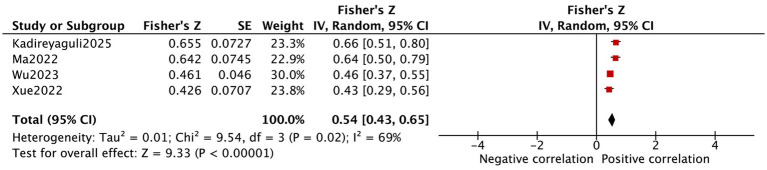
Fisher’s *Z* forest plot for burnout and work–family conflict.

Figure 4 shows the pooled results for the association between burnout and work–family conflict. The pooled Fisher’s *Z* value was 0.54 (95% CI: 0.43 to 0.65), which corresponds to a correlation coefficient of *r* = 0.49 (95% CI: 0.41 to 0.57), indicating a moderate positive association between burnout and work–family conflict.

A random-effects model was applied to synthesize the results, considering the potential methodological and clinical heterogeneity across studies. Heterogeneity analysis showed a moderate level of between-study heterogeneity (*I*^2^ = 69%, *p* = 0.02).

#### 3.3.4 Correlation between burnout and presenteeism

**Figure 5:**
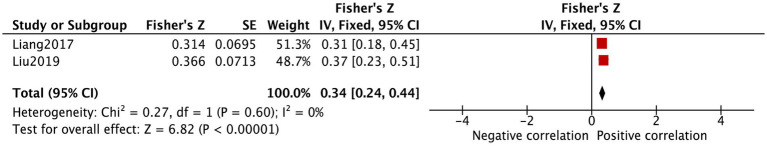
Fisher’s *Z* forest plot for burnout and presenteeism.

Figure 5 shows the pooled results for the association between burnout and presenteeism. The pooled Fisher’s *Z* value was 0.34 (95% CI: 0.24 to 0.44), corresponding to a correlation coefficient of *r* = 0.33 (95% CI: 0.24 to 0.41). These findings indicate a low-to-moderate positive correlation between burnout and presenteeism. No substantial statistical heterogeneity was observed (*I*^2^ = 0%).

#### 3.3.5 Correlation between burnout and turnover intention

**Figure 6:**
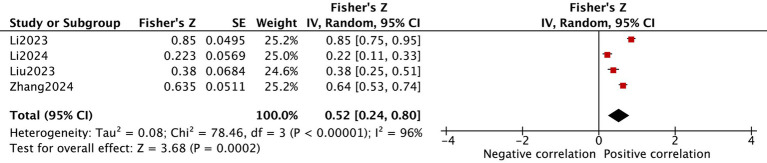
Fisher’s *Z* forest plot for burnout and turnover intention.

Figure 6 shows the pooled results for the association between burnout and turnover intention. The pooled Fisher’s *Z* value was 0.52 (95% CI: 0.24 to 0.80), corresponding to a correlation coefficient of *r* = 0.48 (95% CI: 0.24 to 0.66), indicating a moderate positive association between burnout and turnover intention.

A random-effects model was applied to synthesize the results, considering the potential methodological and clinical heterogeneity across studies. Substantial heterogeneity was observed (*I*^2^ = 96%, *p* < 0.001), suggesting considerable variability across studies.

#### 3.3.6 Correlation between burnout and moral distress

**Figure 7:**
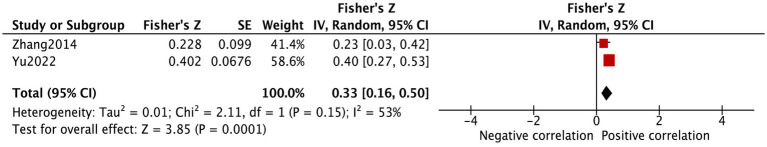
Fisher’s *Z* forest plot for burnout and moral distress.

Figure 7 shows the pooled results for the association between burnout and moral distress. The pooled Fisher’s *Z* value was 0.33 (95% CI: 0.16 to 0.50), corresponding to a correlation coefficient of *r* = 0.32 (95% CI: 0.16 to 0.46), indicating a low-to-moderate positive correlation between burnout and moral distress.

A random-effects model was applied to synthesize the results, considering the potential methodological and clinical heterogeneity across studies. Heterogeneity analysis showed a moderate level of between-study heterogeneity (*I*^2^ = 53%, *p* = 0.15).

#### 3.3.7 Correlation between burnout and alarm fatigue

**Figure 8:**
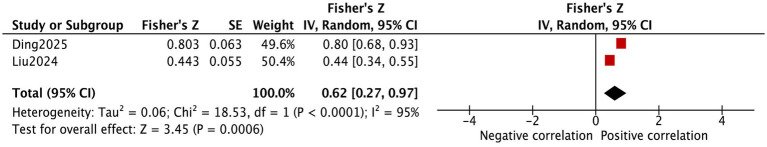
Fisher’s *Z* forest plot for burnout and alarm fatigue.

Figure 8 shows the pooled results for the association between burnout and alarm fatigue. The pooled Fisher’s *Z* value was 0.62 (95% CI: 0.27 to 0.97), corresponding to a correlation coefficient of *r* = 0.55 (95% CI: 0.26 to 0.75), indicating a moderate positive association between burnout and alarm fatigue.

A random-effects model was applied to synthesize the results, considering the potential methodological and clinical heterogeneity across studies. Substantial heterogeneity was observed (*I*^2^ = 95%, *p* < 0.001), suggesting considerable variability across studies.

#### 3.3.8 Correlation between burnout and research competence

**Figure 9:**
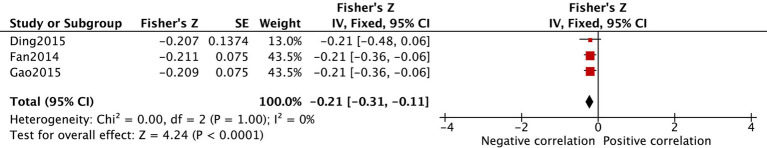
Fisher’s *Z* forest plot for burnout and research competence.

Figure 9 shows the pooled results for the association between burnout and research competence. The pooled Fisher’s *Z* value was −0.21 (95% CI: −0.31 to −0.11), corresponding to a correlation coefficient of *r* = −0.21 (95% CI: −0.30 to −0.11), indicating a low-to-moderate negative association between burnout and research competence.

No substantial statistical heterogeneity was observed (*I*^2^ = 0%).

#### 3.3.9 Publication bias and sensitivity analyses

**Figure 10:**
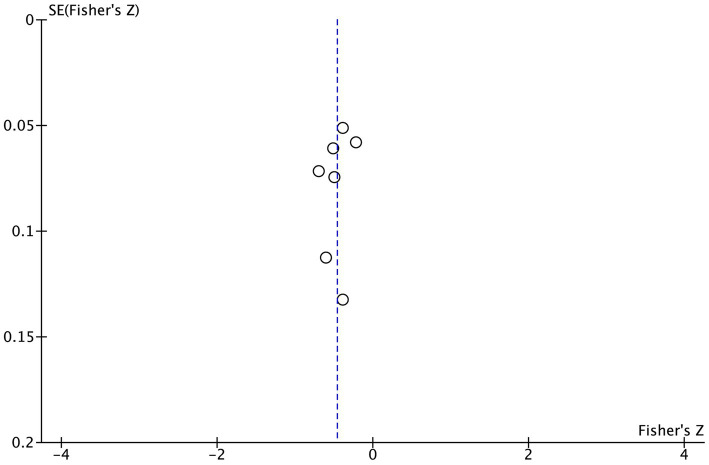
Funnel plot for burnout and psychological capital correlation analysis.

**Figure 11:**
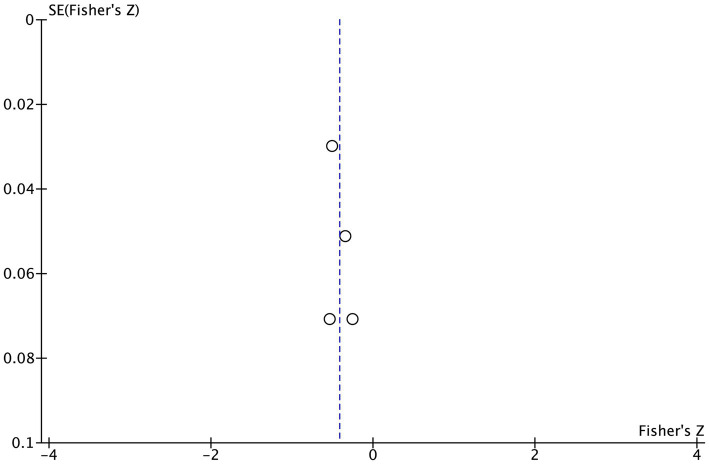
Funnel plot for burnout and social support correlation analysis.

**Figure 12:**
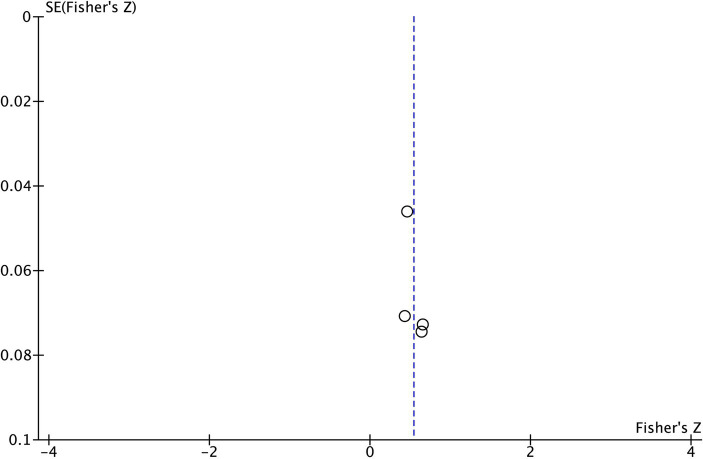
Funnel plot for burnout and work–family conflict correlation analysis.

**Figure 13:**
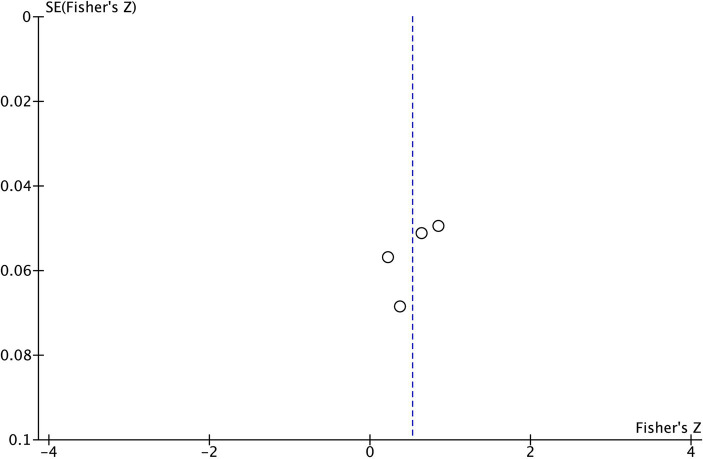
Funnel plot for burnout and turnover intention correlation analysis.

Considering that the number of included studies for some correlates was limited, publication bias and sensitivity analyses were conducted only for factors with a relatively larger number of studies, including psychological capital, work–family conflict, social support, and turnover intention. However, owing to the limited number of studies, the power of funnel plots to detect publication bias may be constrained. As shown in Figures 10–13, the funnel plots for the associations between burnout and psychological capital, social support, work–family conflict, and turnover intention appeared generally symmetrical, with no obvious asymmetry observed.

Sensitivity analyses were conducted using the leave-one-out approach. The results showed that, for the associations between burnout and psychological capital, social support, work–family conflict, and turnover intention, the direction of the pooled effect sizes remained consistent and the magnitude varied only slightly after excluding each study in turn, indicating good robustness of the findings. Detailed results are presented in Tables 5–8.

**Table 5:** Sensitivity analysis of the correlation between burnout and psychological capital.

Program	*Z*-value	95% CI	*I*^2^ (%)
Yao 2022 (9)	−0.44	(−0.58, −0.31)	83%
Jiang 2020 (16)	−0.45	(−0.60, −0.31)	84%
Zheng 2021 (17)	−0.42	(−0.53, −0.31)	72%
Ma 2016 (22)	−0.46	(−0.60, −0.31)	84%
Xia 2019 (23)	−0.51	(−0.61, −0.41)	63%
Zhang 2024 (24)	−0.48	(−0.63, −0.32)	84%
Tang 2020 (25)	−0.47	(−0.61, −0.33)	84%

**Table 6:** Sensitivity analysis of the correlation between burnout and social support.

Program	*Z*-value	95% CI	*I*^2^ (%)
Du 2022 (10)	−0.38	(−0.53, −0.23)	77%
Lin 2017 (18)	−0.38	(−0.53, −0.23)	87%
Xue 2022 (21)	−0.46	(−0.58, −0.35)	77%
Zhang 2024 (24)	−0.44	(−0.59, −0.29)	82%

**Table 7:** Sensitivity analysis of the correlation between burnout and work–family conflict.

Program	*Z*-value	95% CI	*I*^2^ (%)
Wu 2023 (19)	0.57	(0.43, 0.72)	69%
Ma 2022 (20)	0.51	(0.38, 0.63)	68%
Xue 2022 (21)	0.58	(0.44, 0.71)	73%
Kadireyaguli 2025 (44)	0.50	(0.39, 0.62)	62%

**Table 8:** Sensitivity analysis of the correlation between burnout and turnover intention.

Program	Z-value	95% CI	*I*^2^ (%)
Zhang 2024 (24)	0.49	(0.08, 0.89)	97%
Li 2023 (34)	0.41	(0.16, 0.67)	93%
Li 2024 (35)	0.63	(0.37, 0.88)	94%
Liu 2023 (36)	0.57	(0.22, 0.92)	97%

#### 3.3.10 Subgroup analysis

Due to the limited number of studies available for each correlate, subgroup analyses were conducted only for the associations between burnout and psychological capital, social support, work–family conflict, and turnover intention.

For burnout and psychological capital, subgroup analyses were performed according to publication year, region, and sample size. The results suggest potential regional differences, with the correlation appearing numerically stronger among ICU nurses in the northern regions than in the southern regions. However, given the small number of studies included in each subgroup, this finding should be interpreted with caution. No clear influence of publication year or sample size on the correlation was observed (Table 9).

**Table 9:** Subgroup analysis of the correlation between burnout and psychological capital.

Program	Literature volume	Merged *Z*-value	95% CI	Heterogeneity (*I*^2^)
Year				
2016–2019	2	−0.35	[−0.61, −0.08]	88
2020–2022	4	−0.56	[−0.68, −0.44]	47
2024	1	−0.38	[−0.48, −0.28]	−
Region				
South	5	−0.39	[−0.51, −0.28]	73
North	2	−0.66	[−0.78, −0.54]	0
Sample size				
<100	2	−0.50	[−0.70, −0.31]	27
100–299	3	−0.30	[−0.46, −0.14]	79
≥300	2	−0.56	[−0.68, −0.44]	59

For burnout and social support, subgroup analyses were conducted according to publication year, region, and sample size. Because no data were available for the northern subgroup, regional comparisons could not be made. Given the limited number of studies within the subgroups and substantial heterogeneity in some analyses, no definitive subgroup differences can be inferred (Table 10).

**Table 10:** Subgroup analysis of the correlation between burnout and social support.

Program	Literature volume	Merged *Z*-value	95% CI	Heterogeneity (*I*^2^)
Year				
2017	1	−0.54	[−0.68, −0.40]	−
2022	2	−0.39	[−0.63, −0.15]	90
2024	1	−0.34	[−0.44, −0.24]	−
Region				
South	4	−0.42	[−0.54, −0.29]	82
North	−	−	−	−
Sample size				
<300	2	−0.40	[−0.68, −0.12]	87
≥300	2	−0.43	[−0.59, −0.27]	87

Similarly, for burnout and work–family conflict, subgroup analyses based on publication year, region, and sample size indicated a trend toward stronger correlations in more recent publications. Regional differences were also observed. Nevertheless, owing to the limited number of studies within the subgroups, these findings require further confirmation (Table 11). For burnout in relation to turnover intention, although numerical variations in effect sizes were noted across certain subgroups, comparative inferences are not appropriate given the small number of studies included. Furthermore, some subgroups exhibited substantial heterogeneity, and considering the limited number of studies, the stability of these estimates remains uncertain (Table 12).

**Table 11:** Subgroup analysis of the correlation between burnout and work–family conflict.

Program	Literature volume	Merged *Z*-value	95% CI	Heterogeneity (*I*^2^)
Year				
2022	2	0.53	[0.32, 0.74]	77
2023–2025	2	0.55	[0.36, 0.74]	80
Region				
South	2	0.45	[0.37, 0.53]	0
North	2	0.65	[0.55, 0.90]	0
Sample size				
<200	2	0.65	[0.55, 0.75]	0
≥200	2	0.45	[0.37, 0.53]	0

**Table 12:** Subgroup analysis of the correlation between burnout and turnover intention.

Program	Literature volume	Merged *Z*-value	95% CI	Heterogeneity (*I*^2^)
Year				
2023	2	0.62	[0.16, 1.08]	97
2024	2	0.43	[0.03, 0.83]	97
Region				
South	3	0.41	[0.16, 0.67]	93
North	1	0.85	[0.75, 0.95]	−
Sample size				
<300	1	0.38	[0.25, 0.51]	−
≥300	3	0.57	[0.22, 0.92]	97

## 4 Discussion

This meta-analysis showed that psychological capital, social support, and research competence were negatively correlated with burnout, whereas work–family conflict, turnover intention, presenteeism, moral distress, and alarm fatigue were positively correlated with burnout. These findings indicate that burnout among ICU nurses is associated with psychological resources and work-related stressors.

### 4.1 Psychological capital

This study found that psychological capital was moderately negatively correlated with burnout (*r* = −0.43), suggesting that ICU nurses with higher levels of psychological capital tend to have lower levels of burnout. This finding is consistent with previous studies (9, 16, 17, 22–24).

As an important construct in positive psychology, psychological capital includes self-efficacy, hope, resilience, and optimism, reflecting individuals’ positive psychological resources when facing work-related challenges and stress (45). Previous studies have suggested that nurses with higher psychological capital are more likely to adopt adaptive coping strategies and report lower levels of burnout (46).

This finding is supported by international evidence. A recent systematic review and meta-analysis of registered nurses reported a negative association between psychological capital and burnout, with a pooled correlation coefficient similar to that observed in this study. This consistency suggests that psychological capital may be a relevant psychological resource associated with lower burnout across different nursing and healthcare contexts (47). However, the correlation observed in this study was stronger than that reported by Qiao et al. (48), which may be related to differences in study populations and work environments. Because this study did not include comparisons across different work settings, such interpretations require further investigation.

The seven included studies showed a certain degree of heterogeneity, which may be related to differences in ICU type, nurses’ age, and work experience. Sensitivity analysis indicated that the magnitude and direction of the pooled effect size remained stable after sequentially removing individual studies, suggesting acceptable robustness of the results. Additionally, although the funnel plot appeared relatively symmetrical, its ability to detect publication bias was limited due to the small number of included studies.

Subgroup analyses revealed some variation in the pooled effect sizes across publication years and regions. However, given the small number of studies within subgroups and the absence of formal between-group comparisons, these findings should be interpreted as indicative of potential trends rather than definitive differences. Such variations may be influenced by multiple unmeasured factors, and their underlying mechanisms warrant further investigation.

Overall, the negative association between psychological capital and burnout is supported by this study. However, this relationship reflects a statistical association rather than a causal effect. Future research, particularly longitudinal or interventional studies, is needed to further clarify the underlying mechanisms.

### 4.2 Social support

The results of this study showed that social support was negatively correlated with burnout (*r* = −0.40), with a moderate effect size, indicating that higher levels of social support were associated with lower levels of burnout. This finding is consistent with previous studies (10, 18, 21, 24).

Social support can alleviate burnout in high-stress clinical environments by providing emotional support, practical assistance, and a sense of belonging. A systematic review by Velando-Soriano et al. (49) indicated that workplace social support, particularly support from supervisors and colleagues, plays an important role in preventing burnout among nurses. Furthermore, a systematic review and meta-analysis by Quesada-Puga et al. (50) found that ICU nurses with lower job satisfaction had higher levels of burnout, emphasizing the importance of working conditions and work environments in burnout management. These international findings support the present results from the perspectives of both social support and supportive work environments, suggesting that social support is negatively associated with burnout among ICU nurses. However, the sources, forms, and strengths of social support may vary across healthcare systems and cultural contexts.

Four studies were included in this analysis, and substantial heterogeneity was observed, which may be related to differences in measurement tools for social support, work environments, and individual characteristics. Sensitivity analysis conducted by sequentially removing individual studies suggested that the results were relatively robust. Although the funnel plot appeared relatively symmetrical, its ability to detect publication bias may be limited given the small number of included studies.

Subgroup analyses suggested that the pooled effect sizes differed to some extent across publication years, regions, and sample sizes. However, because the number of studies in each subgroup was small and formal between-subgroup comparisons were limited, these findings should be interpreted only as potential trends rather than definitive subgroup differences. Future studies should use more standardized measurement tools, larger multicenter samples, and more refined subgroup designs to clarify the relationship between social support and burnout among ICU nurses.

### 4.3 Work–family conflict

This meta-analysis showed a significant positive correlation between work–family conflict and burnout among ICU nurses (*r* = 0.49), suggesting that ICU nurses with higher levels of work–family conflict tended to report higher levels of burnout. Work–family conflict refers to the stress and conflict that arise when individuals are required to balance work and family roles (51). When such role conflict intensifies, ICU nurses may find it more difficult to balance work and family responsibilities and may be more likely to experience job dissatisfaction and higher levels of burnout.

This finding is broadly consistent with those of studies from regions beyond China. Asiedu et al. (52) reported that family-to-work conflict was positively associated with burnout among registered nurses in Ghana. Blanco-Donoso et al. (53) also found that daily work–family conflict and burnout were associated with higher daily intentions to leave the profession and lower daily vitality among healthcare workers from intensive care and nephrology units. These studies suggest that work–family conflict may be a common burnout-related factor among nurses working in high-stress clinical environments. However, the strength of this association may vary across countries because of differences in staffing levels, shift systems, family responsibilities, and organizational support.

Four studies were included in this analysis, and moderate heterogeneity was observed, which may be related to differences in nurses’ age, family responsibilities, shift patterns, and work environments. Sensitivity analysis showed that the pooled result was relatively stable. Subgroup analysis suggested that the association between work–family conflict and burnout may be stronger among ICU nurses in northern regions than in southern regions. However, because the number of studies in each subgroup was limited and the geographic distribution was uneven, this finding should be interpreted as exploratory. Future studies should include larger samples, cover more diverse regions, and use longitudinal or intervention designs to further examine this relationship.

### 4.4 Presenteeism

The results of this meta-analysis showed that presenteeism was positively correlated with burnout (*r* = 0.33), with a low-to-moderate effect size. This finding is consistent with the results reported by Wang et al. (54), who suggested that higher levels of presenteeism were associated with higher levels of burnout among ICU nurses.

Presenteeism generally refers to a state in which individuals are physically present at work but not fully engaged. Previous studies have suggested that this condition may be associated with reduced work engagement and increased psychological burden and thus may be related to higher levels of burnout. The strength of the association observed in this study was slightly higher than that reported in previous research, which may be related to differences in the study populations or specific work contexts. However, because this study did not include non-ICU comparison groups, direct comparisons across different work environments could not be conducted.

It should be noted that only two studies were included in this meta-analysis, and the current evidence remains limited. Although no substantial statistical heterogeneity was observed (*I*^2^ = 0%), this estimate should be interpreted with caution in the context of a small number of studies. Overall, the association between presenteeism and burnout was preliminarily supported in this study, while its manifestation in different nursing settings and the underlying mechanisms require further investigation.

### 4.5 Turnover intention

The results of this meta-analysis showed that turnover intention was positively correlated with burnout (*r* = 0.48), with a moderate effect size, indicating that ICU nurses with higher levels of turnover intention tend to have higher levels of burnout. This finding is consistent with previous studies (24, 34–36).

Turnover intention is generally regarded as an individual’s subjective evaluation of their current work status, and its development may be influenced by multiple factors, such as work stress, career development opportunities, and organizational support. Previous studies have indicated that, although turnover intention is not equivalent to actual turnover behavior, it may reflect, to some extent, individuals’ adaptation to their work environment and their level of job satisfaction (24). The moderate association observed in this study suggests a close relationship between burnout and turnover intention; however, the direction of this relationship cannot be clearly determined within the current research framework.

A close relationship between burnout and turnover intention has also been reported in other nursing contexts. For example, Turunç et al. (55) found that burnout was significantly associated with turnover intention among ICU nurses in Turkey, whereas Özkan’s meta-analytic review showed that burnout and its main dimensions were related to turnover intention among nurses (56). These findings suggest that burnout may be closely linked to nurses’ intention to leave in different healthcare settings. However, actual turnover is also affected by labor market conditions, salaries, career opportunities, and institutional retention policies. Therefore, the association between burnout and turnover intention should be interpreted not only as an individual psychological response but also as a reflection of broader organizational and workforce management issues.

Four studies were included in this analysis, and heterogeneity was observed, which may be related to differences in measurement tools, study settings, and organizational environments. The sensitivity analysis indicated that the pooled results were relatively stable. Subgroup findings suggested possible differences in the strength of the association under different conditions; however, these results should be interpreted cautiously because of the limited number of studies in some subgroups. Future studies should use longitudinal designs to clarify whether burnout predicts subsequent turnover intention and identify organizational strategies that may reduce both burnout and nurses’ intention to leave.

### 4.6 Moral distress

The results of this study showed that moral distress was positively correlated with burnout (*r* = 0.32), with a low-to-moderate effect size, suggesting that ICU nurses with higher levels of moral distress tend to have higher levels of burnout. Moral distress refers to the psychological conflict and stress experienced when individuals recognize ethically appropriate actions but are constrained from acting in accordance with their moral judgment due to internal or external factors (57). Previous studies have suggested that moral distress is associated with increased psychological burden and higher levels of burnout.

It should be noted that only two studies were included in this analysis; therefore, the current evidence was limited. Additionally, a certain degree of heterogeneity was observed, indicating potential variability across studies. Given the small number of included studies, further subgroup analyses to explore potential influencing factors could not be conducted. Therefore, this association requires further validation in future studies with larger sample sizes.

### 4.7 Alarm fatigue

This study found a moderate positive association between alarm fatigue and burnout among ICU nurses (*r* = 0.55), suggesting that ICU nurses with higher levels of alarm fatigue tend to report higher levels of burnout. Alarm fatigue is a common problem in high-technology ICU environments and is mainly related to nurses’ prolonged and frequent exposure to a large number of clinical alarms, especially false and nonactionable alarms. Repeated alarm exposure may reduce nurses’ sensitivity to alarm signals, increase their psychological burden and work stress, and contribute to higher levels of burnout. Previous research among critical care nurses has also reported an association between alarm fatigue and burnout, suggesting that reducing alarm fatigue may help alleviate burnout among nurses (58).

Additionally, Nyarko et al. (59) reported that alarm fatigue was related to burnout among nurses working in critical care units in Ghana and noted that excessive, false, and nonactionable alarms may contribute to alarm fatigue. This suggests that alarm fatigue is not limited to a specific region but may represent a common occupational stressor across different ICU settings. However, the strength of the association between alarm fatigue and burnout may vary across hospitals owing to differences in monitoring equipment, alarm management policies, alarm-related training, and ICU workflows.

The number of studies included in this analysis was limited, and substantial heterogeneity was observed, which has affected the stability and interpretation of the pooled estimate. Because of the limited number of studies, subgroup and moderator analyses could not be performed. Therefore, this result indicates a correlational trend between alarm fatigue and burnout, and its generalizability across different ICU settings requires further verification. Future studies should include larger samples of nurses from different ICU types and regions and further evaluate whether alarm management optimization, individualized alarm parameter settings, nurse training, and equipment maintenance can reduce alarm fatigue and burnout.

### 4.8 Research competence

The results of this meta-analysis showed that research competence among ICU nurses was negatively correlated with burnout (*r* = −0.21), with a low-to-moderate effect size, suggesting that nurses with higher levels of research competence tend to have lower levels of burnout.

Research competence generally encompasses literature retrieval, study design, data analysis, and academic writing (60), and its development may be influenced by multiple factors, including individual learning engagement, professional development needs, and work status. The negative association observed in this study may reflect that higher levels of burnout are associated with reduced participation in and initiative in research activities, whereas individuals with stronger research competence may have advantages in work adaptation and professional development. However, the direction and underlying pathways of this relationship cannot be clearly determined within the current research framework.

Furthermore, only three studies were included in this analysis, and differences in the study contexts and measurement methods may have affected the stability of the results. Although the heterogeneity analysis indicated *I*^2^ = 0%, in the context of a small number of studies, this estimate has a limited capacity to reflect true consistency. Therefore, the observed association requires further examination in different nursing populations and research settings.

### 4.9 Limitations

This study has several limitations. First, this meta-analysis included only studies reporting Pearson correlation coefficients and total burnout scores to ensure statistical and conceptual consistency; however, this may have reduced the comprehensiveness of the dataset by excluding studies reporting only Spearman coefficients or dimension-level burnout outcomes. Second, all included studies were cross-sectional; therefore, the findings indicate associations only and cannot establish causality or temporal ordering. Third, most studies used convenience sampling, and some analyses included few studies or showed high heterogeneity, which may have affected the generalizability and stability of the results. Future studies should report standardized correlation coefficients, provide both total and dimension-specific burnout outcomes, and adopt longitudinal or interventional designs.

## 5 Conclusion and recommendations

This meta-analysis synthesized evidence on the factors associated with burnout among ICU nurses based on Pearson correlation coefficients. The findings showed that psychological capital, social support, work–family conflict, presenteeism, turnover intention, moral distress, alarm fatigue, and research competence were associated with ICU nurses’ burnout, although the direction and magnitude of these associations varied across factors. For factors reported in fewer than two studies, a quantitative synthesis was not performed; therefore, their relationship with burnout requires further investigation.

Overall, this study provides more integrated and comparable evidence for understanding burnout-related factors among ICU nurses. The findings suggest that burnout among ICU nurses is associated with multiple psychological, occupational, and organizational factors. In clinical nursing management, greater attention should be paid to modifiable factors, such as enhancing psychological capital and social support, reducing work–family conflict and alarm fatigue, and addressing turnover intention and presenteeism. However, the effectiveness of these strategies should be further evaluated through well-designed empirical studies. Future research should use larger samples, longitudinal designs, and multidimensional analyses to clarify the mechanisms underlying ICU nurse burnout and develop targeted intervention strategies.

## Data Availability

The original contributions presented in the study are included in the article/supplementary material, further inquiries can be directed to the corresponding author.
